# Editorial: Epitope Discovery and Synthetic Vaccine Design

**DOI:** 10.3389/fimmu.2018.00826

**Published:** 2018-04-18

**Authors:** Clarisa Beatriz Palatnik-de-Sousa, Irene da Silva Soares, Daniela Santoro Rosa

**Affiliations:** ^1^Laboratório de Biologiae Bioquimica de Leishmania, Microbiologia Geral, Universidade Federal do Rio de Janeiro, Rio de Janeiro, Brazil; ^2^Instituto de Investigação em Imunologia, Faculdade de Medicina, Universidade de São Paulo (USP), São Paulo, Brazil; ^3^Department of Clinical and Toxicological Analyses, Universidade de São Paulo, São Paulo, Brazil; ^4^Departamento de Microbiologia, Imunologia e Parasitologia, Universidade Federal de São Paulo (UNIFESP), São Paulo, Brazil

**Keywords:** vaccines, synthetic vaccines, epitopes, T-epitope vaccines, B-cell epitope vaccines, epitope data-bank, immunoinformatics

**Editorial on the Research Topic**

**Epitope Discovery and Synthetic Vaccine Design**

Traditional and first generation vaccines are composed of live or fixed whole pathogens, while second generation vaccines include, among others, the native protein antigens purified from the pathogen. And furthermore, third-generation vaccines are comprised of DNA plasmids capable of expressing the sequence of the most important pathogen protein antigens in the host. During this evolution of vaccines, there has been a gain in safety, however, with a loss of efficacy that has been compensated for with the use of adjuvants.

The latest step in the evolution of vaccine formulations is the development of epitope vaccines. Epitopes are short amino acid sequences of a protein that can induce a more direct and potent immune response, than the response induced by the whole cognate protein (1).

Moreover, the strategy for developing epitope vaccines requires an accurate knowledge of the amino acid sequence of the immunogenic protein of interest. Therefore, since vaccines against parasite, bacteria, or virus infections and tumors require a cellular immune response for prevention, control, and cure, a strategy called Reverse Vaccinology (RV) was developed. The RV approach uses the information of the codon sequence contained in the DNA of the pathogen to obtain a complementary cDNA, and further translates it to obtain the sequence of the protein of interest. Once these proteins are inside the antigen-presenting cells (APC) of the host, they are processed. The T cell epitopes are then proteolytically cleaved from the protein, and further exposed by the MHC molecules of the APC surface, to interact with the receptors of T cells. Therefore, with the knowledge of the primary sequence of the protein antigen, the epitopes can be identified by cloning the domains or smaller peptides of the protein separately and experimentally determining which one is more immunogenic, or alternatively, by screening the whole protein sequence using *in silico* predictions programs [Fleri et al.; (2, 3)].

The structure of the MHC molecules on APC, MHC class I molecules have a single alpha chain that influences binding, and the binding groove lies between the alpha 1 and alpha 2 domains (Fleri et al.). Since the binding groove is closed, it can only accommodate shorter peptides (8–14 amino acids). The groove binding core has only nine amino acids. MHC class II molecules in contrast, have two chains, alpha and beta that influence binding. The binding groove is open and can accommodate longer peptides (13–25 amino acids) but the binding core has 9 amino acid residues with 0–5 residues flanking on each side. Only the alpha chain is variable in class I molecules, so the nomenclature is “HLA” followed by the locus A, B, or C, an asterisk, and the number of the allele it represents. For class II molecules, both the alpha and beta chains impact binding and both of their chains are variable for the DP and DQ loci. However, for the DR locus, only the beta chain is variable (Fleri et al.). For all the mentioned characteristics, MHC class II binding prediction is more challenging than that for class I molecules. Based on different machine learning algorithms, several predictions were developed as tools to identify the T cell epitopes of the protein antigens [Fleri et al.; (2, 3)].

In contrast, for parasite, viral, bacterial infections and tumors, whose prevention control and cure requires the development of a potent antibody response, the problem is more complex. In fact, the majority of B cell epitopes are discontinuous epitopes composed of amino acid residues located on separate regions of the protein, and which are joined together by the folding of the chain (4). These groups of residues cannot be isolated as such from the antigen. Therefore, the strategy used for these cases is called structural based reverse vaccinology (SBRV), and it focuses on the use of monoclonal antibodies against the protein antigen. There are six complementary determining regions (4) or antigen-binding regions (ABRs) (5), in the antibody molecule that can interact with the antigen. An antigen-binding site, also called a paratope, which is a small region (of 10–15 amino acids) is the part of the antibody that recognizes and binds to an antigen. However, each ABR differs significantly in its amino acid composition and tends to bind different types of amino acids on the surface of proteins. In spite of these differences, the combined preference of the six ABRs does not allow the epitopes to be distinguished from the rest of the protein surface. These findings explain the poor success of past and newly proposed methods for predicting protein epitopes (4, 5). SBVR strategy is used to study the interaction of the complex composed of the monoclonal antibody with the protein in order to identify to which amino acids of the antigen protein, the ABR or paratope of the monoclonal antibody binds. The objective of this approach is to elucidate the potential amino acid sequence of the discontinuous epitope indirectly. However, the search for the epitopes that interact with antibodies is a much more difficult task to which successful prediction algorithms are about non-existing. Consequently, this strategy has not achieved much success (4, 5).

The inability of synthetic linear peptides to effectively mimic the discontinuous epitopes is one of the reasons for the failure of many B cell synthetic vaccines to induce the synthesis of neutralizing antibodies. These facts partially explain why even though more than a thousand synthetic B cell peptides have been identified, only 125 of them have progressed to phase I, 30 of them to Phase II, and none of them have succeeded in phase III trials or have been licensed for human use (4).

Hence, while RV generally refers to the *in silico* analysis of the entire pathogen genome to identify all the antigens that the pathogen is able to express, the SBRV refers to the approach which tries to generate a vaccine from the known crystallographic structure of the neutralizing antibodies bound to the epitopes (6).

In the case of infections that are preventable by an antibody response, the term antigenicity has frequently been confused with immunogenicity (7). In fact, the epitopes of some viral antigens are often wrongly considered as immunogens, when they are only antigens, since they can interact with a variety of antibodies raised against a virus, but they are not capable of inducing the synthesis of the neutralizing antibodies engaged in protection (7). Previously, it was thought, that if an antigenic epitope bound strongly to a neutralizing monoclonal antibody *in vitro*, it would be also able to induce the synthesis of neutralizing antibodies when used as a vaccine. However, this is not true (7).

Additionally, other concepts have been developed in association to the RV strategy (6). The concept of RV 1.0 is an approach based on bioinformatics and animal immunization and challenge used to determine which antigens are more appropriate for vaccination (8). In contrast, the concept of RV 2.0 refers to a strategy that obtains monoclonal antibodies from the few individuals that make a strong antibody response against natural infection. These monoclonal antibodies guide the vaccine design in the reversal direction of the normal flow of vaccines to anti-bodies (8).

Furthermore, the concept of “rational vaccine design” was used very often creating the expectation of having the same success as the strategy of “rational drug design” obtained before. However, the “rational drug design” is related to the development of chemical analogs that are perfect inhibitors of the active site of important vital enzymes of the pathogen. In contrast, investigators involved in the development of the HIV vaccine claimed that they use the “rational vaccine design” whereas in fact they only improved the antigenic binding capacity of one epitope with respect to only one paratope, and not the immunogenic capacity of an epitope to elicit neutralizing antibodies. These conclusions generated strong criticism [Van Regenmortel; (9)].

In contrast, the present Research Topic uses the concept of “Epitope Discovery and Synthetic Vaccine Design” as illustrated by Kao and Hodges (1). These authors demonstrated that synthetic vaccines based on short peptides, which represent immunogenic epitopes are able to impair and even exceeded the protective potential of the native cognate whole protein. They found higher antibody titers directed to the receptor-binding domain of the Pilus A of *Pseudomonas aeruginosa*, which has 14 amino acids than to the whole pilin native protein. The titers against the native pilin of the animals immunized with the synthetic peptide-conjugate were higher, than the titers of animals immunized with the whole pilin protein. In addition, the affinities of the anti-peptide sera for the intact pilin receptor-binding domain were significantly higher than affinities of anti-pilin protein sera (1).

We support the development of epitope vaccines that combine immunoinformatics and experimental biological approaches (Alves-Silva et al.; Barbosa Santos et al.). We used an immunoinformatic approach to improve the efficacy of existing vaccines composed of protein antigens that were selected according to their relevance in previous experimental biological results. Our results also showed that vaccines composed of the immunogenic domains, optimize and even exceed the protective potential induced by the whole protein (1). For instance, we achieved 33% optimization of vaccine efficacy by using a recombinant chimera, which contains the two domains that hold the most immunogenic epitopes of the Nucleoside hydrolase NH36 of *Leishmania*, instead of the whole NH36 protein (Alves-Silva et al.). These two domains (F1 and F3) hold the most potent epitopes for the generation of prophylactic protection against *Leishmania (L.) amazonensis* infection (Alves-Silva et al.). Vaccination with the NH36 protein reduces the lesion sizes by 55% (10). However, vaccination with the F1 and the F3 domains independently determined respective reductions of 70 and 77%, and the F1F3 chimera induced a reduction of 82% in the footpad lesion sizes (Alves-Silva et al.).

This enthusiasm coming after the advent of immunoinformatic tools and the finding of epitopes *via in silico* predictions should not devalue the empirical foundations of all experimental science involved in the development of the vaccines that control diseases until present (6). On the contrary, both the empirical and *in silico* tools should be used together in the development of new synthetic epitope vaccines that offer advantages over traditional vaccines. They are chemically defined antigens free from deleterious effects. Additionally, in contrast to live-attenuated vaccines, they do not revert to virulence in immunocompromised subjects, and different from genetic vaccines, they do not involve ethical questions.

With this Research Topic, we believed we have made significant contributions to the development of synthetic epitope vaccines that may help in the prevention, treatment, and control of infectious diseases and cancer.

## Author Contributions

CP-d-S, DSR, and ISS wrote and approved the final text of this Editorial.

## Conflict of Interest Statement

The authors declare that the research was conducted in the absence of any commercial or financial relationships that could be construed as a potential conflict of interest.
